# Enlightened *Mannhemia haemolytica* lung inflammation in bovinized mice

**DOI:** 10.1186/1297-9716-45-8

**Published:** 2014-01-25

**Authors:** Fabio Franco Stellari, Sophia Lavrentiadou, Francesca Ruscitti, Sarah Jacca, Valentina Franceschi, Maurizio Civelli, Chiara Carnini, Gino Villetti, Gaetano Donofrio

**Affiliations:** 1 Department of Medical Veterinary Science, University of Parma, via del Taglio 10, 43126 Parma, Italy; 2 Corporate Pre-clinical R&D, Chiesi Farmaceutici S.p.A, Largo Francesco Belloli 11/A, 43122 Parma, Italy; 3 Department of Biomedical Biotechnological and Translational Sciences, University of Parma, via del Taglio 10, 43126 Parma, Italy

## Abstract

Polymorphonuclear cells diapedesis has an important contribution to the induced *Mannhemia haemolytica* (*M. haemolytica*) infection lung inflammation and IL-8 is the primary polymorphonuclear chemoattractant. Using a bovine IL-8/luciferase transiently transgenized mouse model, the orchestration among *M. haemolytica*, IL-8 promoter activation and neutrophilia was followed in real time by in vivo image analysis.

## Introduction, methods and results

*Mannheimia haemolytica*, called in the past *Pasteurella haemolytica*, is the pathogenic bacteria mostly associated with respiratory diseases in ruminants, and ruminant pneumonic pasteurellosis is a valuable large animal model for human pneumonia. Pneumonic pasteurellosis is a fibronecrotising pneumonia with extensive infiltration of small airway and alveoli with polymorphonuclear (PMN) inflammatory cells [1,2]. Among PMN cells, neutrophils are the major contributors to the lung parenchyma damage [3] and the neutrophil factors contributing to the lung tissue injury have been identified and can be listed: elastases, acid hydrolases, oxidative radicals, complement proteins, cytokines and chemokines. The recruitment of neutrophils to the injured tissue is orchestrated by chemokines, and IL-8 (interleukin 8 or CXCL) is the most remarkable one. A significant correlation between IL-8 levels and neutrophil infiltration in diseases has been reported. IL-8 is an essential factor for acute inflammation and induces the infiltration of T lymphocytes into inflamed tissue.

In recent studies [4] it was hypothesized that although mouse cells do not bear a clear homologous to IL-8 gene, the murine transcriptional apparatus could nonetheless be capable of activating or repressing a heterologous IL-8 gene promoter driving a reporter gene. The possibility of monitoring in vivo, in small rodents, luciferase reporter genes under the control of cytokine promoters, derived from other species, is of great value. This allow to study the pathophysiology of inflammatory responses, as well as to test interventions aimed at modulating these responses. In fact, the above hypothesis was successfully tested by a molecular imaging approach utilizing a previously well-characterized bovine IL-8 promoter/luciferase [5] transiently transgenized (bovinized) mouse model [4]. *M. haemolytica* products, such as leukotoxin and lipopolysaccharides, are considered triggering factors of the inflammatory response and the consequent lung injury. In the present note, taking advantage of the in vivo imaging of the IL-8 bovinized mouse model [4], the direct correlation among *M. haemolytica* inflammatory products, bovine IL-8 activation and neutrophilia was investigated.

Initially, nine 7-8 weeks-old female inbred FVB mice (Harlan Laboratories, San Pietro al Natisone, Udine, Italy) were transiently transgenized with pbIL-8-luc [5], a reporter construct generated by sub-cloning the 2030 bp IL-8 promoter in front of a pGL3 Luciferase reporter vector. Briefly, 40 μg of plasmid constructs DNA and 7 μL of JetPEI were each diluted into 200 μL 5% glucose. The two solutions were then mixed and incubated for 15 min at room temperature. The entire mixture was intra-venously injected (tail vein) into FVB mice and the expression of luciferase was monitored through in vivo imaging with an IVIS imaging system (Caliper Life Sciences, Alameda, CA, USA).

**Figure 1:**
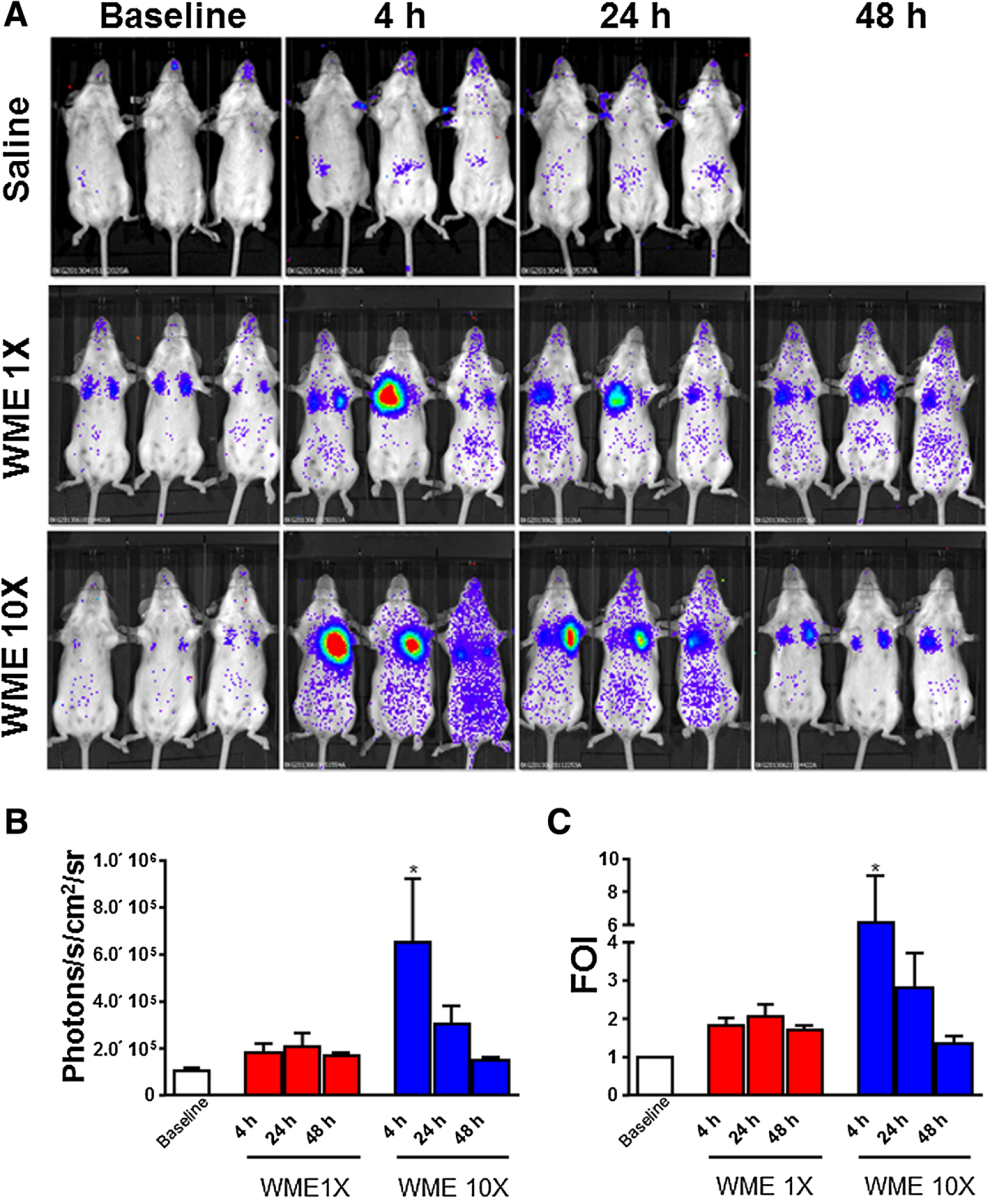
**Bovinized mice are responsive to WME. (A)** Representative image of pbIL-8-Luc bovinized mice treated with saline (*n* = 3), WME 1X or WME 10X. Mice were monitored before the treatment to get the baseline and at 4, 24 and 48 h post treatment by in vivo image analysis drawing a region of interest (ROI) over the chest and using an IVIS imaging system (Caliper Life Sciences, Alameda, CA). Light emitted was acquired from specific regions by Living Image® software (Caliper Life Sciences, Alameda, CA) and expressed as photon/second/cm^2^ (photon/s/cm^2^) **(B)** and normalized as fold of induction versus baseline (FOI Vs. Baseline) **(C)**. Statistical differences were tested by One Way ANOVA followed by Dunnet’s post hoc test for group comparisons. Results are reported as mean ± SD and significance attributed when *P* < 0.05 (*) or *P* < 0.01 (**).

*M. haemolytica* (reference strain NTCT 9380, Serotype 2, ATCC 33396) was grown at 37 °C with aeration on BHI agar supplemented with NAD and hemin-solution (stock-solution containing a mixture of hemin, l-histidine, and triethanolamine) or in BHI broth without NAD and hemin supplementation [6]. The whole *M. haemolytica* extract (WME) was prepared as described by Roier et al. [7] (10X; corresponding to 20 μg of total protein content and 1X; corresponding to 2 μg of total protein content). Ten days post transgenization, 3 mice were intratracheally instilled with 50 μL of PBS, 3 mice with 50 μL of WME 10X and 3 mice with 50 μL 1X WME. Mice were monitored for luciferase expression in the lung by in vivo image analysis at different time (4, 24 and 48 h) post-instillation. Mice specifically responded to WME at 4 h post treatments, especially when treated with the higher dose (10X) of WME. The response remained significant after 24 h (Figure 1), while no signal was observed in control animals treated with saline (Figure 1).

Further, a positive control was established with mice transiently transgenized with pNFKB-luc (pGL4.32[luc2P/NF-kB-RE/Hygro]; Promega), a responsive promoter containing nuclear factor-kappaB (NF-*k*B) binding sites (Additional file 1). These above experiments were repeated twice and similar results were obtained with a statistical significance. All animals were acclimatized for at least 5 days to the local vivarium conditions (room temperature: 20–24 °C; relative humidity: 40–70%), having free access to standard mouse chow and tap water. All experiments were carried out in rodents and exclusively included painless suppression of animals. The experiments comply with the Principles of Animal Care (publication no. 85–23, revised 1985) of the National Institutes of Health and with the current law of the European Union and Italy (D. L.vo 116/92). The present project was approved by the Ethical Committee of the University of Parma (Italy).

**Figure 2:**
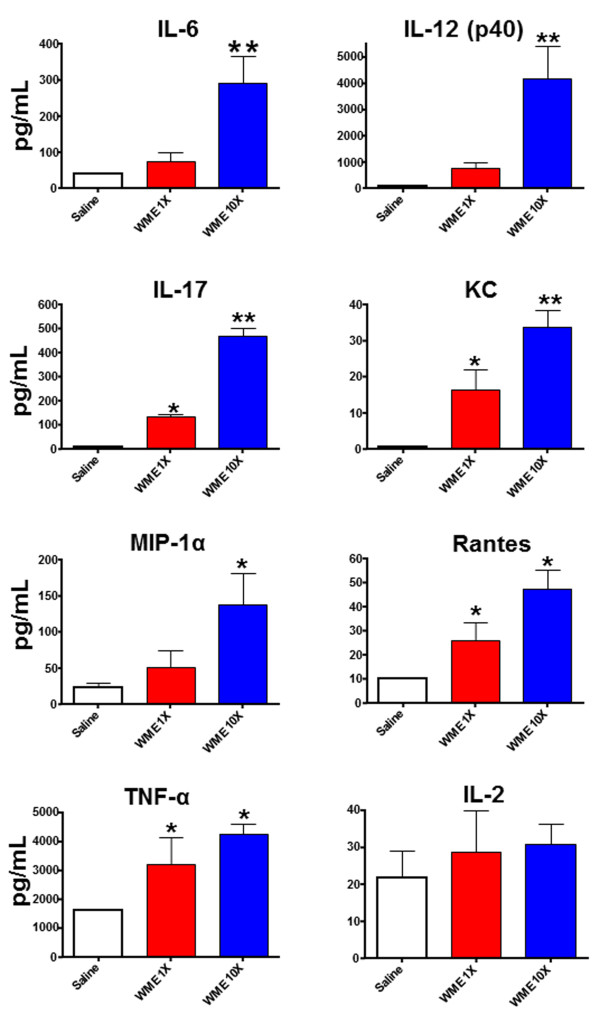
**Impact of WME on cytokines expression.** Pattern of up-regulated cytokines in the BAL of WME 1X or WME 10X intra-tracheally treated pbIL-8-Luc bovinized mice in respect to the saline-treated control group. Results are reported as mean ± SD and significance attributed when *P* < 0.05 (*) or *P* < 0.01 (**).

**Figure 3:**
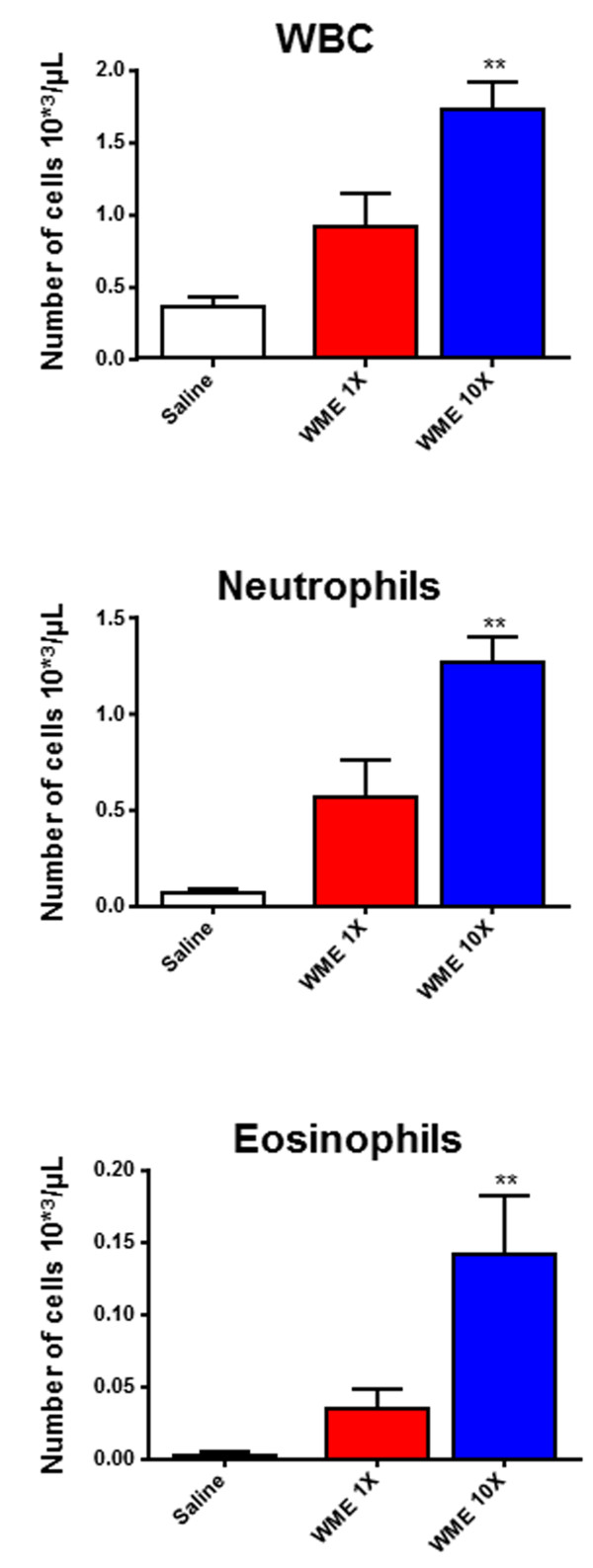
**Impact of WME on WBC infiltration.** Cellular infiltration into the lung of mice intratracheally instilled with WME 1X, WME 10X or vehicle. The amount of White blood cells (WBC), Neutrophils and Eosinophils found in BALF was expressed as number of cells per μL as measured by Dasit Sysmec XT 1800. Results are reported as mean ± SD and significance attributed when *P* < 0.05 (*) or *P* < 0.01 (**).

Although a specific response was shown, in term of luciferase reporter gene transcription, following WME administration, this phenomenon could be merely confined to the transcriptional level without having something to do with downstream pathological events such as cytokine storms, PMN cell infiltration and neutrophilia. To further explore this, an identical group of mice was bovinized and WME-treated. At 4 h post-treatment, which was the time corresponding to the maximal bIL-8-luc activation, their lungs were intratracheally washed and the broncho-alveolar lavage fluid (BALF) was assayed for the presence of cytokines, infiltrating total white blood cells (WBC) and neutrophils. Interleukin 6 (IL-6), the p40 subunit of interleukin 12 (IL-12p40), chemokine ligand 1 (KC or CXCL1), macrophage inflammatory protein 1 alpha (MIP-1α), chemokine ligand 5 [(CCL5 or Regulated on Activation, Normal T cell Expressed and Secreted (RANTES)] and tumor necrosis factor-alpha (TNF-α) were measured by Luminex cytokine assays (Bio plex 200 system, Bio Rad; Bio-Plex Pro™ Mouse Cytokine Group I) as suggested by the company. IL-6, IL-12p40, KC, MIP-1α, RANTES and TNF-α, but not IL-2, significantly increased following WME treatment in respect to the untreated control (Saline) (Figure 2). Moreover, the increase in cytokine and chemokine production was accompanied by a strong increase in white blood cells (WBC), among which the neutrophils were the most abundant (Figure 3). WBC phenotyping (Additional file 2) was performed by Flow cytometry [FACS Canto II, 2 lasers, 6 colors, Becton Dikinson. Antibodies: anti mouse CD 45 PE-Cy5, BD Pharmigen; anti mouse F4/80 Alexa 488 (AbD Serotec); anti mouse CD11b PE-Cy7 (BD Pharmigen); anti mouse Lys6G (BD Pharmigen)], whereas cell count was done by Dasit Sysmec XT 1800.

## Discussion

IL-8 is a peptide secreted by different immune and non-immune cells. The most important function of IL-8 is to chemo attract neutrophils [8] as well as T-cells [9] and monocytes [10]. IL-8 secretion is a secondary event, dependent on the prior secretion of early response molecules such as IL-1β and TNF-α [11-13]. Physiologically IL-8 is barely detectable, but it is rapidly transcriptionally induced and its transcript stabilized in response to pathological processes, as in the case in *M. haemolytica* lung infection in ruminants [1,2]. A variety of signaling pathways regulate IL-8 transcription and mRNA stabilization and these include: nuclear factor-kappaB (NF-kB) and p38 mitogen activate protein kinase [14,15].

With these information in mind, it was of interest to investigate if IL-8 transcriptional activation could be a valuable molecular read-out to visualize *M. haemolytica* infection induced lung inflammation in vivo, using the mouse as animal model, although pneumonic pasteurellosis is a typical disease of ruminants. Since lung diseases manifestation in ruminants overlap with the majority of human lung diseases manifestation, this model could be of great value for human lung diseases too. Moreover, the use of ruminants as animal model is very costly and demanding in terms of maintenance and the availability of advanced genetic or immunologic resources is significantly limited when compared with those available for rodent studies.

In this work the functionality of a bovine IL-8/luciferase reporter construct was successfully tested in mice, taking advantage of a bioluminescent imaging (BLI) approach. The capability of BLI to monitor in real time a biological process, longitudinally in the same animal in vivo, represents an obvious advantage for functional as well as pathological and pharmacological studies. In fact, because mammalian tissues do not naturally emit bioluminescence, a specific signal could be collected when *M. haemolytica* WME was intra-tracheally administrated to the lung of transietly trangenized mice with a bovine IL-8/luciferase reporter construct. Further, the increase of the bioluminescence signal was correlated with an increase of WBC and cytokines. Noteworthy, although mice do not have an IL-8 gene, mouse cell signaling and their transcriptional apparatus could specifically activate the bovine IL-8 gene promoter; KC, a mouse cytokine homologous to IL-8, was one of the cytokines to be up-regulated too.The correlation between high WBC content, neutrophilia and luciferase expression following WME stimulation is in accordance with the recognized IL-8 role as a key factor in orchestrating inflammation, this further validates the association between IL-8 transcriptional activation and pulmonary inflammation in the experimental setting that we adopted. Although a single isolate of *M. haemolytica* has been employed in this report, this paves the way to the use of other *M. haemolytica* isolates or other bacterial species, which could be easily compared in terms of inflammatory response and thus, a direct correlation between isolate, inflammatory response and pathogenicity can be easily achieved.

## Competing interests

The authors declare that they have no competing interests.

## Authors’ contributions

FFS: performed the experiments. SL, FR, SJ, and VF: contribute to the experiments. MC, CC and GV: intellectually contributed. GD: conceived the experiments, contribute to the experiments and wrote the paper. All authors read and approved the final manuscript.

## Supplementary Material

Additional file 1 **pNFKB-luc transiently transgenized mice response to WME.** Representative image of mice transiently transgenized with pNF-kB Luc and treated with saline (*n* = 3), WME 1X or WME 10X. Mice were monitored before the treatment to get the baseline and at 4, 24 and 48 h post treatment by in vivo image analysis drawing a region of interest (ROI) over the chest and using an IVIS imaging system (Caliper Life Sciences, Alameda, CA, USA). Light emitted was acquired from specific regions by Living Image® software (Caliper Life Sciences, Alameda, CA, USA) and expressed as photon/second/cm^2^ (photon/s/cm^2^) and normalized as fold of induction versus baseline (FOI). Statistical differences were tested by One Way ANOVA followed by Dunnet’s post hoc test for group comparisons. Results are reported as mean ± SD and significance attributed when *P* < 0.05 (*) or *P* < 0.01 (**). Click here for file

Additional file 2 **BAL WBC immune-phenotyping.** Bronchoalveolar lavage was performed and cells were subsequently analyzed for surface markers. A) Forward and side scatter plots showed an increase in cell recruitment compared to saline. Gating was performed on CD45 positive cells to discard debris and on F4/80 negative cells to differentiate granulocyte population from macrophages. B) Gating was performed of neutrophil-specific surface markers CD11b and GR-1 and their upregulation correlated with the higher dose of WME. Click here for file
