# Supplementary figures and images for: Enlightened Mannhemia haemolytica lung inflammation in bovinized mice
Source: Vet Res. 2014 Jan 25;45(1):8. doi: 10.1186/1297-9716-45-8 (PMC3906860; doi:10.1186/1297-9716-45-8)

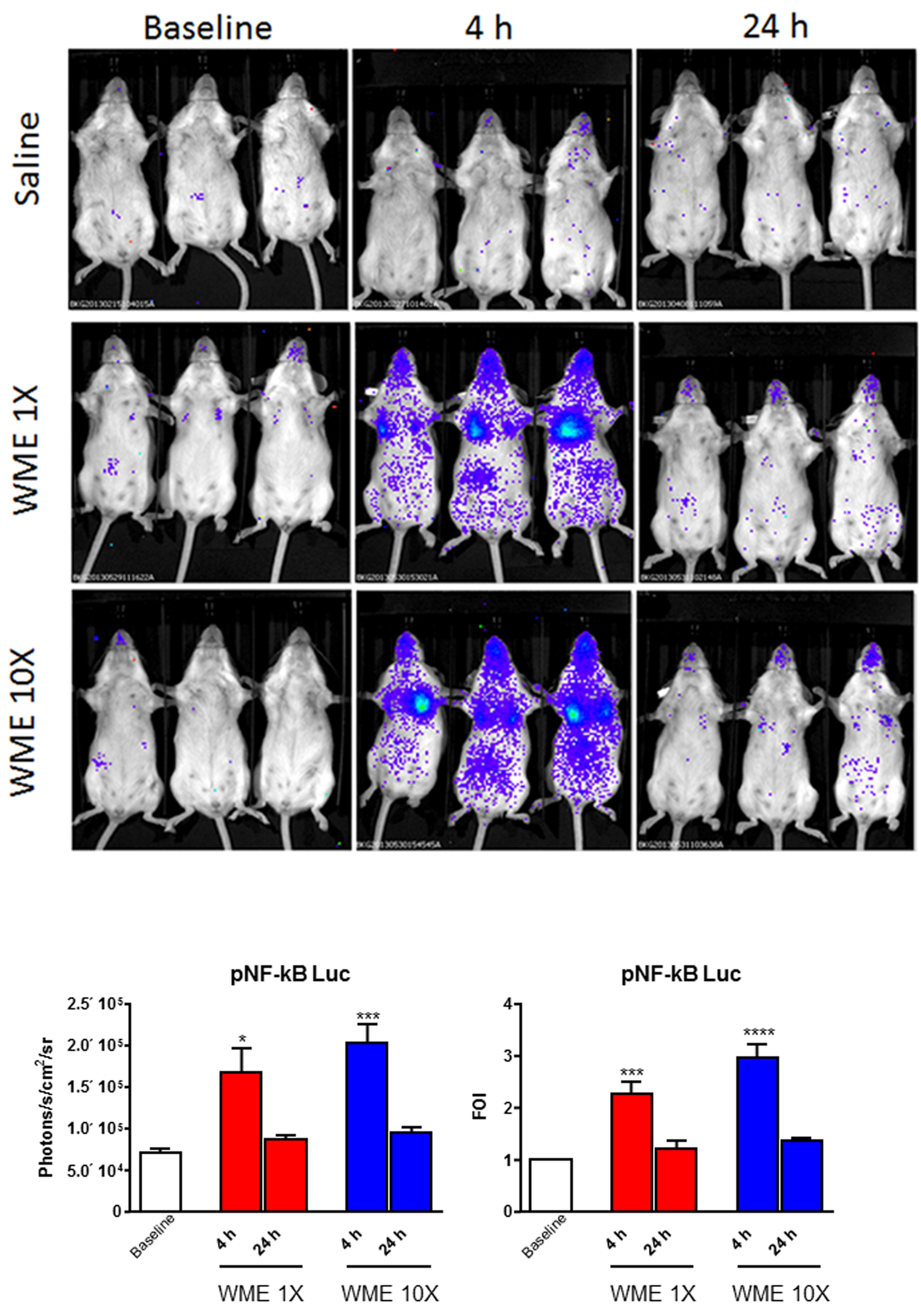

Supplement: Additional file 1 — pNFKB-luc transiently transgenized mice response to WME. Representative image of mice transiently transgenized with pNF-kB Luc and treated with saline (n = 3), WME 1X or WME 10X. Mice were monitored before the treatment to get the baseline and at 4, 24 and 48 h post treatment by in vivo image analysis drawing a region of interest (ROI) over the chest and using an IVIS imaging system (Caliper Life Sciences, Alameda, CA, USA). Light emitted was acquired from specific regions by Living Image® software (Caliper Life Sciences, Alameda, CA, USA) and expressed as photon/second/cm2 (photon/s/cm2) and normalized as fold of induction versus baseline (FOI). Statistical differences were tested by One Way ANOVA followed by Dunnet’s post hoc test for group comparisons. Results are reported as mean ± SD and significance attributed when P < 0.05 (*) or P < 0.01 (**). [file 1297-9716-45-8-S1.tiff]

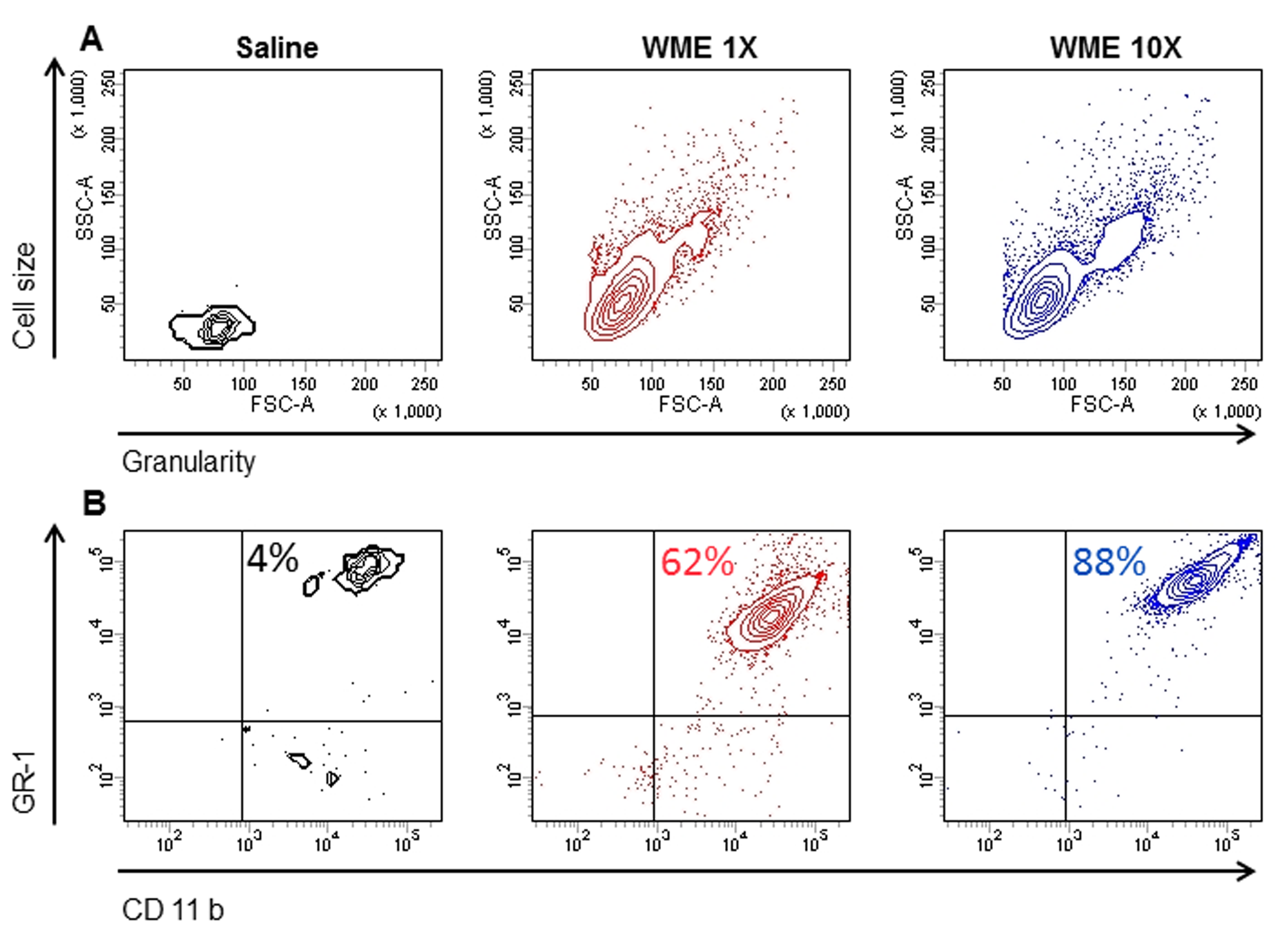

Supplement: Additional file 2 — BAL WBC immune-phenotyping. Bronchoalveolar lavage was performed and cells were subsequently analyzed for surface markers. A) Forward and side scatter plots showed an increase in cell recruitment compared to saline. Gating was performed on CD45 positive cells to discard debris and on F4/80 negative cells to differentiate granulocyte population from macrophages. B) Gating was performed of neutrophil-specific surface markers CD11b and GR-1 and their upregulation correlated with the higher dose of WME. [file 1297-9716-45-8-S2.tiff]
